# Carbon-Emission Characteristics and Influencing Factors in Growing and Shrinking Cities: Evidence from 280 Chinese Cities

**DOI:** 10.3390/ijerph19042120

**Published:** 2022-02-14

**Authors:** Xinhua Tong, Shurui Guo, Haiyan Duan, Zhiyuan Duan, Chang Gao, Wu Chen

**Affiliations:** 1 Northeast Asian Studies College, Jilin University, Changchun 130012, China; tongxinhua@jlu.edu.cn (X.T.); guosr20@mails.jlu.edu.cn (S.G.); changgao20@mails.jlu.edu.cn (C.G.); 2 College of New Energy and Environment, Jilin University, Changchun 130012, China; 3 Auditing Office of Jinniu, Chengdu 610036, China; chenwu19@mails.jlu.edu.cn

**Keywords:** CO_2_ emissions, growing cities, shrinking cities, STIRPAT, comprehensive index, China

## Abstract

The CO_2_ emission-mitigation policies adopted in different Chinese cities are important for achieving national emission-mitigation targets. China faces enormous inequalities in terms of regional economic development and urbanization, with some cities growing rapidly, while others are shrinking. This study selects 280 cities in China and divides them into two groups of growing cities and two groups of shrinking cities. This is achieved using an index called “urban development degree,” which is calculated based on economic, demographic, social, and land-use indicators. Then, the 280 cities’ CO_2_ emission characteristics are examined, and extended STIRPAT (stochastic impacts by regression on population, affluence, and technology) is used to verify the influencing factors. We find that rapidly growing cities (RGCs) present a trend of fluctuating growth in CO_2_ emissions, rapidly shrinking cities (RSCs) show an inverted U-shaped trend, and slightly growing (SGCs) and slightly shrinking cities (SSCs) show a trend of rising first, followed by steady development. Moreover, for growing cities, the population, economy, and proportion of tertiary industry have positive effects on carbon emissions, while technology has negative effects. For shrinking cities, the population and economy have significant positive effects on carbon emissions, while technology and the proportion of tertiary industry have negative effects.

## 1. Introduction

China has become one of the world’s major consumers of energy and emitters of carbon dioxide. The Chinese government has therefore set a goal to reach peak carbon emissions by 2030 and carbon neutrality by 2060 [1,2]. Cities, as highly concentrated places of population and industry, typically produce about 80% of total carbon emissions; in Chinese cities, the proportion is as high as 85% [3]. Therefore, controlling urban carbon emissions is crucial for achieving emission-reduction targets [4,5]. In China, with rapid urbanization and enormous imbalances in regional development, some cities are growing, while others are shrinking [6,7]. The populations and economies of growing and shrinking cities are varied, resulting in divergent effects on carbon emissions. Accordingly, CO_2_ emission-mitigation policies need to be adjusted according to the characteristics of different cities [5]. It is necessary, therefore, to explore the characteristics and driving factors of carbon emissions in growing and shrinking cities to more effectively achieve emission targets.

## 2. Literature Review

Urban carbon-emission trends, peak forecasting, and the related influencing factors have attracted considerable research attention. Regarding carbon-emission trends, the research clearly indicates that emissions are on the rise in China. Zhou et al. [8], for example, found that carbon emissions increased continuously from 1992 to 2013 in all Chinese cities, growing faster in eastern China than in central and western China. Zhu et al. [9] found that carbon emissions doubled from 1997 to 2012 in Tianjin. However, while total carbon emissions have shown an overall growth trend, emissions are decreasing in certain sectors. For instance, using multiscale input–output analysis, Hung et al. [10] found that carbon emissions in Hong Kong’s construction industry decreased from 2004 to 2011. Meanwhile, with China’s stated aim to reach carbon peak by 2030, many studies have assessed its feasibility, arriving at two opposing views: “can achieve” and “cannot achieve.” For example, using data for 50 Chinese cities, Wang et al. [11] predicted that emissions in those cities would peak between 2021 and 2025. Huang et al. [12] similarly found that under existing policies, Guangzhou would reach peak carbon in 2023. However, Lin et al. [13] found that the limited use of clean energy and ongoing rapid economic growth in Xiamen would cause it to reach carbon peak later than the 2030 target. Likewise, Zhang et al. [14] conducted three simulations on the timing of peak carbon in Baoding, and two of the scenarios indicated that peak carbon would not be achieved until 2040.

The factors affecting carbon emissions are another important area investigated in the research. Many studies have shown that socioeconomic factors, such as economic development, population growth, technology, industrial structure, and energy structure, have important effects on urban carbon emissions [8,15,16,17]. Generally speaking, economic development and population growth increase the demand for energy, resulting in an increase in carbon emissions. Ou et al. [18], for example, studied the socioeconomic factors affecting carbon emissions in cities with different developmental levels in China and found that economic and population growth increased emissions in cities at all levels. Taking 128 countries as samples, Dong et al. [19] found that countries with larger populations consumed more energy and thus generated more carbon emissions. Economic growth, however, does not always increase emissions. When economic development reaches a certain level, carbon emissions will decline; that is, the economy has an inverted U-shaped effect on carbon emissions. Studying 276 large cities around the world, Fujii et al. [20], for example, found that emissions first rose and then decreased with economic growth. Technological progress and industrial structure optimization typically improve energy efficiency and reduce emissions. Wang et al. [21], for instance, found that technology was negatively correlated with carbon emissions. Meanwhile, Li et al. [22] suggested that reducing the proportion of secondary industry and prioritizing the development of tertiary industry could be beneficial for reducing emissions in Chinese cities. Optimizing the energy mix means increasing the use of clean energy, which directly leads to a decrease in carbon emissions. Boluk et al. [23], for example, found that electricity generation using renewable energy helped to improve environmental conditions in Turkey. Similarly, Xu et al. [24] suggested that if China slows down its energy consumption and shifts toward low-carbon fuels, its emission targets could be feasible. Although the abovementioned socioeconomic factors affect carbon emissions, the effects are different. Economic development and population growth tend to increase emissions, while technological progress and industrial and energy structure optimization can decrease emissions.

The existing research tends to study all cities together without distinguishing between them. In fact, with ongoing economic development and population mobility, cities currently face two different development states: growth and shrinkage [25,26]. Different cities have different characteristics in terms of economies and population, and their effects on the environment are also different [27,28,29]. Studies have confirmed that growing and shrinking cities exhibit different energy-efficiency and carbon-emission characteristics. For example, after classifying growing and shrinking cities based on a population index, Xiao et al. [30] found that emissions in rapidly shrinking cities presented a continuously increasing trend, while growing cities reached their emission peaks during 2011–2013. Liu et al. [31] also used a population index to study emissions in growing and shrinking cities and found that urban shrinkage increased emissions and that the energy efficiency of shrinking cities was lower than that of growing ones.

Our review of the literature reveals limitations in the existing research. First, although many studies have focused on the characteristics of and factors affecting urban carbon emissions, they tend to ignore the potential differences between different types of cities. Second, although some studies have comparatively investigated the emissions of shrinking and growing cities, the classifications of those cities are mostly based on a single population index, lacking comprehensive classification. In truth, in addition to population changes, urban growth and shrinkage also involve economic, social, and land-use factors [32]. In light of the above, this study constructs an index, called “urban development degree,” using economic, demographic, social, and land-use indicators. Then, cities are divided into growing and shrinking cities, and their carbon-emission characteristics and related influencing factors are investigated. The findings can provide a reference for emission-mitigation policies.

There are three contributions of this paper. First of all, we focus our research on the city level rather than the national or provincial level, which is a further complement to the micro-level research on carbon emissions. Secondly, we divide different types of urban development patterns by using a comprehensive indicator calculated from socioeconomic indicators, rather than researching all cities under a unified framework. Thirdly, we conduct an in-depth analysis of the influencing factors of carbon emissions in different types of cities, and analyze the reasons for the differences in carbon emissions, so as to provide a targeted reference for the formulation of carbon emission reduction policies and low-carbon development paths for cities with similar characteristics.

## 3. Method

### 3.1. Research Framework

**Figure 1:**
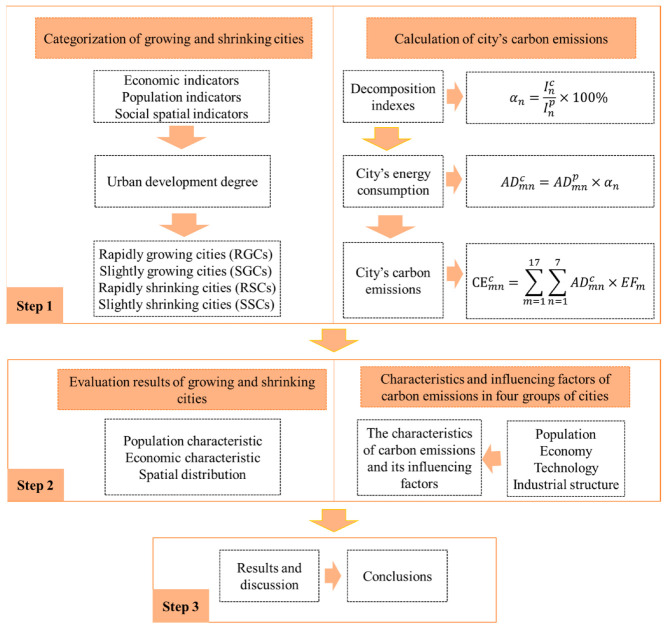
Research framework.

Figure 1 presents a schematic framework of this study’s methodological approach. The framework consists of three parts. The first divides the growing and shrinking city groups and calculates their carbon emissions. The second part compares the social development and carbon-emission characteristics of four groups of cities and uses extended STIRPAT (stochastic impacts by regression on population, affluence, and technology) to test the factors affecting carbon emission. Finally, the third part involves discussing the results and presenting the conclusions.

### 3.2. Categorization of Shrinking and Growing Cities

There are two ways to classify growing and shrinking cities. One is to consider the change in population over a certain period, where an increase in population is identified as urban growth, and a decrease is identified as urban shrinkage [6,28,33]. The other way is to consider the change in nighttime light over a certain period; when nighttime light brightens, the city is growing, and when it dims, the city is shrinking [34,35,36]. However, population division can only reflect changes in the urban population, and while nighttime light can reflect the economy and population, the data are not continuous in time and are characterized by uncertainty, which will affect the results. Therefore, some scholars try to use a comprehensive index to identify growing and shrinking cities. For example, Lin et al. [37] believe that urban growth and shrinkage are characterized by the changes in economy, population, land use and finances. Then, they construct a comprehensive index called urban development degree by using total population, economic growth, employment and unemployment and built-up area data to evaluate growing and shrinking cities in China. Zhang et al. [38] determine that the growth and shrinkage of cities are characterized by the changes in population, economy and social consumption, and shrinking cities often face economic downturns, shrinking population and declining spending power. Referring to Lin et al. [37] and Zhang et al. [38], this study, therefore, constructs an index of urban development degree (UDD) to divide growing and shrinking cities.

Calculating UDD requires the following demographic, economic, and social indicators: (1) Population includes three indicators, natural population growth rate, total population, and population density. Natural population growth rate refers to the difference between birth rate and death rate, which represents the change in natural population growth. Total population refers to the registered urban population, representing the change in the total population. Population density refers to the number of people per unit of land, representing the change in population density. (2) The economy includes per capita GDP (gross domestic product), per capita fiscal revenue, and GDP growth rate. Per capita GDP is the output level of unit population, and per capita fiscal income is the fiscal income of unit population, representing the level of urban economic development. GDP growth rate is the percentage increase in urban output, representing the speed of economic development. (3) The social and land-use dimensions include three indicators: total retail sales of consumer goods, per capita fiscal expenditure, and built-up area. Total retail sales of consumer goods represent a city’s consumption capacity. Per capita fiscal expenditure is the level of fiscal expenditure per unit of population, representing the government’s service capacity. Built-up area refers to the actual developed area in a city, representing the spatial change in urban land use. See Table 1 for details.

Equations (1)–(8) show the calculation process for UDD.

When $X_{\mathrm{ij}}\;$is positive,

(1) $$X_{\mathrm{ij}}^{’}=\frac{X_{\mathrm{ij}}-{\mathrm{minX}}_{j}}{{\mathrm{maxX}}_{j}-{\mathrm{minX}}_{j}},$$

When $X_{\mathrm{ij}}\;$is negative,

(2) $$X_{\mathrm{ij}}^{’}=\frac{{\mathrm{maxX}}_{j}-X_{\mathrm{ij}}}{{\mathrm{maxX}}_{j}-{\mathrm{minX}}_{j}},$$

(3) $$Y_{\mathrm{ij}}=X_{\mathrm{ij}}^{’}/\sum_{i=1}^{m}X_{\mathrm{ij}}^{’},$$

(4) $$e_{j}=-k\sum_{i=1}^{m}(Y_{\mathrm{ij}}\times {\mathrm{lnY}}_{\mathrm{ij}}),\;k=\frac{1}{\ln\left(m\right)},$$

(5) $$d_{j}=1-e_{j},$$

(6) $$W_{j}=d_{j}/\sum_{j=1}^{n}d_{j},$$

(7) $${\mathrm{index}}_{\mathrm{it}}=\sum_{j=1}^{n}(W_{j}\times X_{\mathrm{ij}}^{’}),$$

(8) $${\mathrm{UDD}}_{\left({\mathrm{it}}_{0,}{\mathrm{it}}_{1}\right)}={\mathrm{index}}_{{\mathrm{it}}_{1}}-{\mathrm{index}}_{{\mathrm{it}}_{0}},$$

where i represents the city, j represents the indicator, t represents the year, n represents the number of indicators, and m represents the number of cities. Equations (1) and (2) are the process for standardizing the original data. $X_{\mathrm{ij}}$ and $X_{\mathrm{ij}}^{’}\;$represent the original data and standardized data of the j index of city I, respectively. Equations (3)–(6) show the process for calculating the index weight; $W_{j}\;$is the index weight. Equations (7) and (8) show the process for calculating UDD. ${\mathrm{index}}_{\mathrm{it}}\;$represents the urban development index, and ${\mathrm{UDD}}_{\left({\mathrm{it}}_{0,}{\mathrm{it}}_{1}\right)}$ represents UDD from $t_{0}$ to $t_{1}$.

Following the literature on growing and shrinking cities [6,28], we identify a city whose ${\mathrm{UDD}}_{\left({\mathrm{it}}_{0,}{\mathrm{it}}_{1}\right)}<0$ as a shrinking city and a city whose ${\mathrm{UDD}}_{\left({\mathrm{it}}_{0,}{\mathrm{it}}_{1}\right)}\geq 0$ as a growing city. In addition, based on research on group divisions and the development characteristics of urban populations and economies [29], we divide the cities into the following four groups: (1) rapidly growing cities (RGCs), UDD≥0.05, which have rapid population, economic, and consumption development; (2) slightly growing cities (SGCs), 0≤UDD<0.05, in which the population increases and the economy and consumption develop steadily; (3) rapidly shrinking cities (RSCs), UDD<−0.02, which show sharp declines in population, economy, and consumption; and (4) slightly shrinking cities (SSCs), −0.02≤UDD<0, which are characterized by population decreases and slow growth in consumption and the economy.

### 3.3. CO_2_ Emission Accounting

Since carbon emissions from fossil fuel consumption account for more than 90% of total carbon emissions, this study only calculates carbon emissions from urban fossil-energy consumption. In addition, in the absence of an urban energy balance table in China, following Shan et al. [39,40], we scale down the provincial energy balance table to the city level based on GDP, demographic data, and industrial output. The calculation of CO_2_ follows the Intergovernmental Panel on Climate Change (IPCC). The calculation formula for carbon emissions is as follows:

(9) $${\mathrm{CE}}_{\mathrm{mn}}^{c}=\sum_{m=1}^{17}\sum_{n=1}^{7}{\mathrm{AD}}_{\mathrm{mn}}^{c}\times {\mathrm{EF}}_{m},$$

where${\;\mathrm{CE}}_{\mathrm{mn}}^{c}$ represents CO_2_ emissions from 17 fuel types, m is the energy type, n represents the main industry sector, ${\mathrm{AD}}_{\mathrm{mn}}^{c}$ is the consumption of m fuel in sector n, and ${\mathrm{EF}}_{m}$ is the emission factor. The default IPCC emission factors are used in this study.

### 3.4. STIRPAT Model

STIRPAT is widely used to examine the factors affecting CO_2_ emissions. It is based on Ehrich and Holdren’s (1971) IPAT (impact of population, affluence, and technology) model and has been widely used to examine the effects of human activity on environmental change. The STIRPAT formula is as follows:

(10) $$I={\mathrm{aP}}^{b}A^{c}T^{d}\varepsilon ,$$

where I is environmental impact, P is population, A is affluence, and T is technology level. a is the dominant factor; b, c, and d are the parameters to be estimated; and ε denotes the random error. Taking the logarithm form to both sides, the STIRPAT model can be expressed as below:

(11) lnI=lna+blnP+clnA+dlnT+lnε,

where ln () is the natural logarithm, and b, c, and d are equivalent to elasticity coefficients in economics, which can be regarded as the percentage change in the environment caused by a 1% change in one influencing factor under the condition that other factors remain unchanged.

The STIRPAT model allows additional explanatory factors to be added. In this study, referring to Cai et al. [16] and Wang et al. [41], we augment STIRPAT by adding industrial structure. The augmented model is given as

(12) $$\mathrm{lnI}=\mathrm{lna}+\beta_{1}{\mathrm{lnP}}_{\mathrm{it}}+\beta_{2}{\mathrm{lnA}}_{\mathrm{it}}+\beta_{3}{\mathrm{lnT}}_{\mathrm{it}}+\beta_{4}{\mathrm{lnQ}}_{\mathrm{it}}+\ln\varepsilon ,$$

where I represents CO_2_ emissions, a is the intercept term, P is the total population, and T is the technological level, which is represented by the reciprocal of energy intensity and is obtained by the ratio of GDP to energy consumption. Q is the industrial structure, represented by the proportion of output value of tertiary industry; $\beta_{1},\;\beta_{2},\;\beta_{3},\;\mathrm{and}\;\beta_{4}$ are the elastic coefficients of population, economy, technology, and industry structure, respectively.

In this study, the panel data method based on extended STIRPAT model was used to empirically investigate the relationship between influencing factors and CO_2_ emissions. The panel data analysis is a statistical method to analyze two-dimensional observations collected from multiple entities over multiple times. Compared with conventional models using only time-series or cross-sectional data, this method has the advantages of providing more degrees of freedom and reducing the effects of multi-collinearity [18]. Three models are commonly used in panel data analysis: mixed-effects model, fixed-effects model and random-effects model. F-test and Hausman test are required to determine which model to use for regression. We conduct regressions on the four groups of cities to test whether there are differences in the factors affecting carbon emissions in different groups. After using the F-test and Hausman test on the four groups, a panel fixed-effect model was selected for regression (see Section 4.3 for the empirical results).

### 3.5. Research Objects and Data Sources

Research objects: The research object of this paper involves cities at the prefecture level and above. Due to the lack of data in some cities, 280 cities were finally identified, and their economy and population accounted for more than 80% of China’s. At the same time, they cover all provinces in China and are widely distributed.

Data sources: The data used to construct UDD (i.e., registered population, population density, natural population growth rate, per capita GDP, per capita fiscal expenditure, GDP growth rate, built-up area, population fiscal expenditure, and total retail sales of consumer goods) are from The Statistical Yearbook of Chinese Cities, covering the two years of 2010 and 2019. The GDP of each industry and population used for scaling are derived from cities and their corresponding provincial statistical yearbooks from 2010 to 2019. The province energy balance tables are from the China Energy Statistical Yearbook for 2010–2019. Missing population density data for 2018 were calculated from the ratio of the registered population to land area, which was obtained from the China Urban Statistical Yearbook. Some missing data are supplemented with data for adjacent years.

## 4. Results and Discussion

### 4.1. Results for Growing and Shrinking Cities

**Figure 2:**
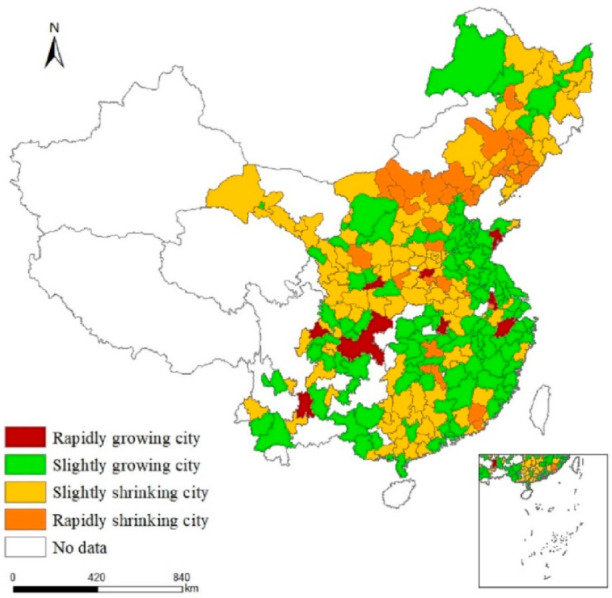
Spatial distribution of shrinking and growing cities in China, 2009–2018.

Based on the value of UDD and the categorization rules, 145 cities shrank during 2009–2018, accounting for 51.79%, mostly distributed in northeastern, central, and western China. A total of 135 cities grew, accounting for 48.21%, mostly distributed in the eastern coastal areas (Figure 2). Among the growing cities, 126 had slight growth, and 9 had rapid growth, accounting for 45% and 3.21%, respectively. Among the shrinking cities, 33 were rapidly shrinking, and 112 were slightly shrinking, accounting for 11.79% and 40%, respectively. Cities with slight growth account for the highest proportion, while those with rapid growth account for the lowest proportion. Most cities in China are SGCs or SSCs, while only a few are RGCs or RSCs.

#### 4.1.1. Characteristics of Growing Cities

**Figure 3:**
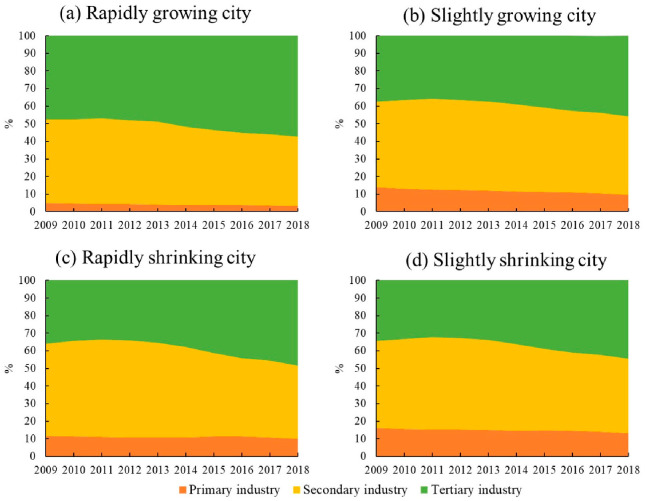
Industrial structure of growing and shrinking cities.

The UDD of RGCs exceeds 0.05; these include Nanjing, Hangzhou, Zhengzhou, Wuhan, Xi’an, Chongqing, Chengdu, Kunming, and Qingdao. Most of these cities are provincial capitals with convenient transportation, good medical conditions, and high levels of education. Their economic development is at the highest level, with per capita GDPs of 50,000–110,000 yuan, and their economies are rapidly expanding. All of these RGCs have populations close to 10 million and are growing rapidly. Moreover, these cities have completed their industrial transformation and upgrading. In 2018, tertiary industry accounted for more than 55% of all industry in these cities (Figure 3a).

The UDD of SGCs ranges from 0 to 0.05; examples include Jinan, Suzhou, Ningbo, Jiaxing, Xiamen, and Fuzhou, mostly distributed in Shandong, Jiangsu, Zhejiang, and Fujian Provinces. Most of these cities are economically developed provincial capitals, with per capita GDPs of 40,000–80,000 yuan. Their economic development levels are lower than those of RGCs, but their economic growth is steady. Furthermore, the SGCs have established a leading position in tertiary industry by optimizing their industrial structures, with the proportion of tertiary industry accounting for more than 45% of the total (Figure 3b). This means that the service industry is constantly improving in these cities. Finally, their total populations show a trend of steady growth.

#### 4.1.2. Characteristics of Shrinking Cities

The UDD of RSCs is below −0.02. Examples include Anshan, Fushun, Zhangjiakou, Tangshan, Baotou, and Daqing, which are mostly distributed in Liaoning, Hebei, and Inner Mongolia. Most of these cities are resource based, mainly relying on mineral resources during their early stage of development, with secondary industry accounting for more than 50% of the total (Figure 3c). Adjustments to the industrial structure started late in these cities. Their economic development is below the middle level, with per capita GDPs mostly between 30,000 and 60,000 yuan. RSCs are experiencing economic decline and population loss.

The UDD of SSCs is between −0.02 and 0. This includes Yichun, Liaoyuan, Datong, Kaifeng, Zigong, and other cities, mostly distributed in Heilongjiang, Jilin, Shanxi, Henan, Gansu, and Sichuan Provinces. Compared with RSCs, SSCs are mostly resource-based cities, taking secondary industry as the leading industry. After industrial restructuring, however, secondary industry accounted for less than 50%, while the service industry accounted for nearly 45% (Figure 3d). In addition, these cities are far away from economically developed cities, have poor geographical location conditions and transportation accessibility, and have relatively low levels of economic development. Their per capita GDP is 20,000–50,000 yuan. Their economies are growing but their populations are diminishing.

### 4.2. Emission Characteristics of Growing and Shrinking Cities

**Figure 4:**
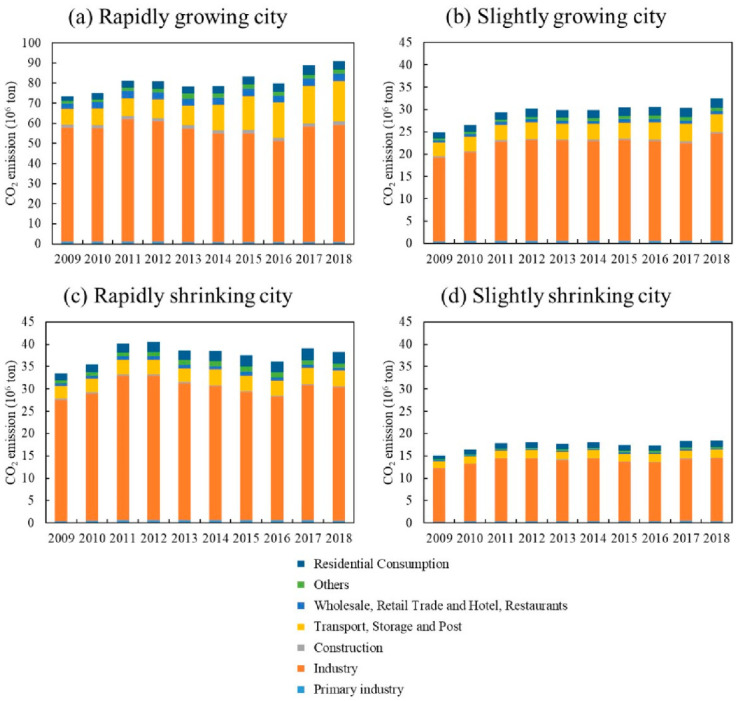
CO_2_ emission characteristics of growing and shrinking cities.

The carbon emissions of RGCs show a trend of fluctuating growth, RSCs present an inverted U-shaped trend, and SGCs and SSCs rise first and then develop steadily (Figure 4). The carbon-emission trends of the four groups are different, and their driving factors may be different as well. Here, we analyze the possible effects of economies, industrial structure, and technology on the carbon-emission characteristics of the four city groups.

#### 4.2.1. Rapidly Growing Cities (RGCs)

**Figure 5:**
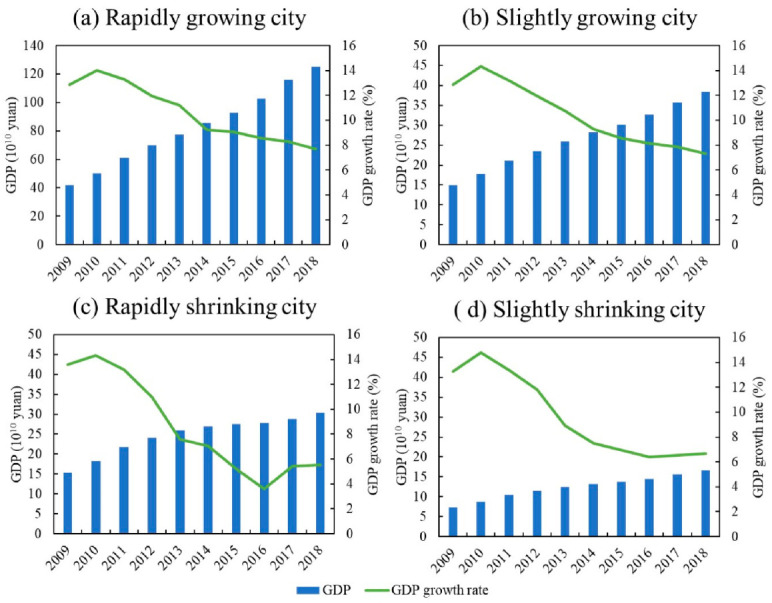
GDP and GDP growth rate of the four city groups.

**Figure 6:**
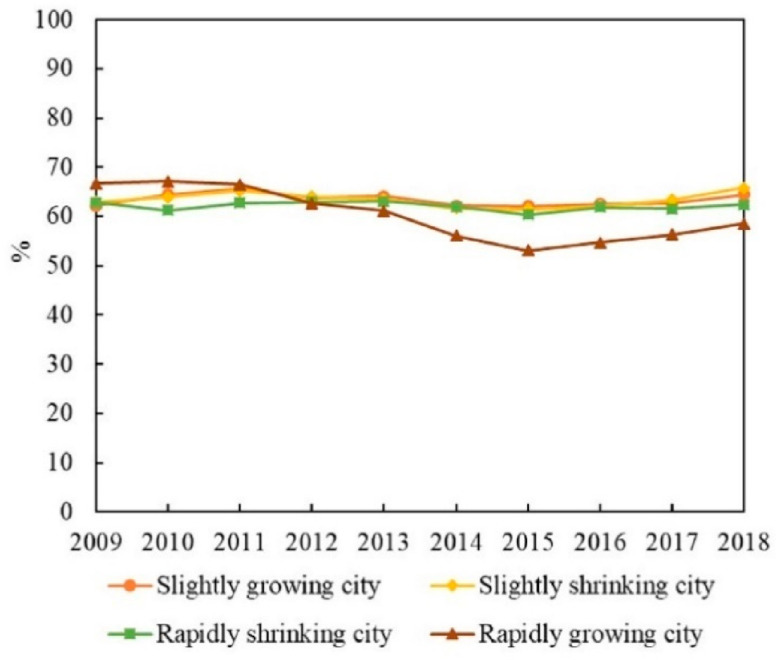
Coal share of growing and shrinking cities.

**Figure 7:**
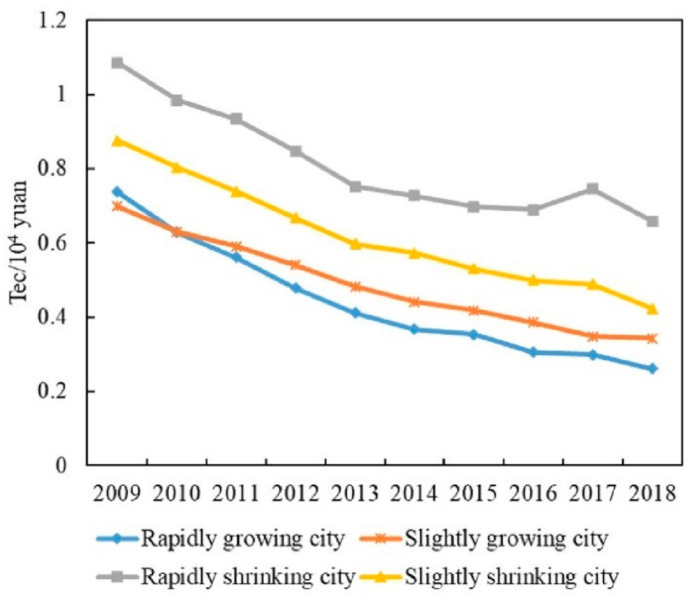
Energy intensity of growing and shrinking cities.

The carbon emissions of RGCs are the highest among the four groups, with a trend of fluctuating growth (Figure 4a). From 2009 to 2012, carbon emissions grew at an annual rate of 3.3%. They fluctuated from 2013 to 2016 and continued to grow at an average annual rate of 2.4% in 2017 and 2018. In terms of economic growth, the GDP of RGCs shows an increase of 298.9%, with primary, secondary, and tertiary industries increasing by 202.9%, 247.6%, and 361.7%, respectively. This indicates rapid economic expansion, which contributes to increases in emissions. Between 2013 and 2016, however, the growth rate of GDP slowed down (annual GDP growth during this period was around 10%, lower than in other periods) (Figure 5a), which slowed the growth of carbon emissions over the period. In terms of industrial structure, the proportion of primary and secondary industry continues to decline, while the proportion of tertiary industry continues to rise. The service industry in RGCs is relatively mature and has occupied a dominant position for a long time, making increasing contributions to the economy. Therefore, the development of services may contribute to the increase in carbon emissions. In terms of energy structure, the proportion of coal in RGCs generally shows a downward trend, especially during 2012–2015. The proportion of coal shows a rapid decline (Figure 6), which may help to reduce carbon emissions. In terms of technology, energy intensity drops from 0.74 to less than 0.26 (tec/10^4^ yuan), an annual decrease of about 11%, showing a rapid downward trend (Figure 7). This means that technology was greatly improved during the study period. We can see that the energy structure and technology of RGCs have been optimized and improved, which helps reduce carbon emissions. However, the carbon-increasing effect of rapid economic expansion and expanded tertiary industry is greater than the carbon-reducing effect of energy structure optimization and technological progress. Therefore, overall urban carbon emissions show an increasing trend.

#### 4.2.2. Slightly Growing Cities (SGCs)

The carbon emissions of SGCs are low among the four city groups, showing a trend of first rising and then changing only slightly (Figure 4b). Specifically, from 2009 to 2012, the carbon emissions of SGCs increased from 24.83 million tons to 30.18 million tons, with an annual growth rate of 5%. Between 2013 and 2018, emissions were around 30 million tons, a small change. In economic terms, the economies of SGCs show an increase of 258.2%. However, GDP growth generally shows a downward trend after 2012 (Figure 5b). Although economic expansion may increase carbon emissions, a decline in economic growth might also slow down the growth of emissions, accounting for the slowed growth of emissions after 2012. In terms of industrial structure, the proportion of primary and secondary industry continues to decline, while that of tertiary industry keeps rising. Especially after 2012, the proportion of secondary industry dropped rapidly (annual decline of 2%). This means that the cities reduced their dependence on energy-intensive industries, which helps reduce carbon emissions. In terms of energy structure, the proportion of coal in SGCs is 62%–66%, and there is a small decline (Figure 6). The urban energy structure has been optimized, and the decline in the proportion of coal is conducive to reducing carbon emissions. In terms of technology, the energy intensity of SGCs decreases year by year, from 0.7 in 2009 to 0.34 (tec/10^4^ yuan) in 2018, an annual decrease of 7.6% (Figure 7). This could be another reason for the slowed growth of carbon emissions. In general, economic expansion in the SGCs increased carbon emissions. After 2012, however, with slowed economic growth, accelerated changes in industrial structure, energy structure optimization, and technological progress, the growth of carbon emissions was restrained, stabilizing changes in emissions.

#### 4.2.3. Rapidly Shrinking Cities (RSCs)

Among the four city groups, RSCs’ carbon emissions are high, presenting an inverted U-shaped trend; 2013 is a turning-point year (Figure 4c). Regarding the economy, despite RSC economies showing growth, their GDP growth rate first rises and then declines rapidly, indicating that their economies declined after a period of growth (Figure 5c). This is consistent with the change characteristics of carbon emissions. We can infer, therefore, that economic changes triggered the changes in emissions. In terms of industrial structure, the proportion of secondary industry increases first and then decreases, while tertiary industry shows the opposite trend. This means that the cities gradually reduced their dependence on secondary industry and developed tertiary industry, which could curb carbon emission growth. In terms of energy structure, the overall change in coal consumption is relatively small (Figure 6), which differs greatly from the change in carbon emissions. Therefore, energy structure might not have an effect on carbon emissions. Meanwhile, the energy intensity of RSCs decreases from 0.88 to 0.42 (tec/10^4^ yuan) (Figure 7); technology was improved, which is conducive to reducing carbon emissions. This analysis reveals that economic and industrial structure (secondary industry) and carbon emissions show the same change characteristics; thus, they may be the main reasons for changes in emissions. Although technological progress can restrain carbon emissions, emissions still show an increasing trend in the early stage. Therefore, technological progress had a weak effect on reducing emissions in the early part of the study period but might account for the reduced emissions in the latter part.

#### 4.2.4. Slightly Shrinking Cities (SSCs)

SSCs have the lowest total carbon emissions among the four city groups. Their carbon-emission trend is similar to that of SGCs, showing a trend of first rising and then changing only a little (Figure 4d). Specifically, carbon emissions rose from 15.14 million tons to 18.09 million tons from 2009 to 2012 and then remained around 18 million tons. In terms of economic development, the GDP of SSCs expanded overall, but the GDP growth rate declined year by year, especially after 2012 (Figure 5d). In terms of industrial structure, the change characteristics for SSCs are similar to those of RSCs, with the proportion of secondary industry first rising and then falling and that of tertiary industry first falling and then rising. Specifically, the proportion of secondary industry rose from 49.3% in 2009 to 51.16% in 2013, and then dropped to 42.18% in 2018. The proportion of tertiary industry fell from 34.5% to 32.8% and then rose to 44.6%. The expansion of economic scale increased carbon emissions, but the slowdown of economic growth and the adjustment of industrial structure after 2012 helped to curb emissions, making them relatively stable. In terms of energy structure, the proportion of coal in SSCs increased from 62.9% in 2009 to 65.8% in 2018 (Figure 6); this means that the energy structure did not improve. Therefore, the energy structure might not be the reason for reduced carbon-emission growth. The energy intensity of SSCs declined annually by 5.4% (Figure 7). The development of low-carbon technologies might be the reason for the reduced growth of emissions.

### 4.3. Regression Results

According to the F-test and Hausman test results, for SGCs, RSCs and SSCs, the fixed-effects model should be used for regression, while the mixed-effects model should be used for RGCs, as shown in Table 2. However, by comparing the mixed-effects regression results with the fixed-effects regression results, the significance of the coefficients of influencing factors has not changed a lot. Therefore, in order to be comparable with the regression results for the other three groups of cities, a fixed-effects model was used for RGCs.

Table 3 shows the estimation results of the fixed-effects model for four city groups. It can be found that the regression results of the models in SGCs, RSCs and SSCs perform better, showing that the R-squared values are more significant. The regression result for RGCs is relatively insignificant, mainly due to the small samples, which makes its explanatory power limited. However, the regression result for RGCs is similar to SGCs, mainly because they belong to growing cities, so the results are reasonable to a certain extent.

Based on the regression results, population and economic growth have a positive effect on carbon emissions, while technological progress has a negative effect on carbon emissions. The proportion of tertiary industry has a positive impact on growing cities, but has a negative impact on shrinking cities. For RGCs, the proportion of tertiary industry has the largest and most significant impact on emissions, which means that the proportion of tertiary industry is the primary influencing factor for carbon emissions in RGCs. An increase of 1% in the proportion of tertiary industry increases carbon emissions by 1.337%. For SGCs and RSCs, population is the most important factor affecting carbon emissions, showing that carbon emissions increase by 0.943% and 0.722% for every 1% increase in population. The technological progress has the greatest negative impact on SGCs, RSCs and SSCs. For every 1% increase in technological level, carbon emissions decrease by 0.709%, 0.518% and 0.547%. For SSCs, the economic growth has the largest positive impact, as every 1% increase in the economy increases carbon emissions by 0.74%.

In order to further verify the robustness of the regression results, we run a Stochastic Frontier Analysis (SFA) model on the extended STIRPAT, and the SFA results are in high agreement with the regression results, indicating our result is robust. Moreover, referring to Wang et al. [21], we compare the fitting data of CO_2_ emission (lnCO_2′_) calculated by the regressions coefficients in Table 3 with the actual CO_2_ emissions (lnCO_2_). The margin of error is within 10%, and most are less than 5%, as shown in Table 4, indicating that the regression model is available.

### 4.4. Discussion

In China, slightly growing and slightly shrinking cities account for the majority, while rapidly growing and rapidly shrinking ones account for a small proportion. Growing cities are mostly distributed in the eastern region and shrinking cities in the northeastern, central, and western regions. This is consistent with Yang et al. [36]. After China’s “reform and opening up,” the eastern region attracted an inflow of FDI (foreign direct investment) by virtue of geographical advantage. This created many jobs, and many laborers came there in search of work, prompting rapid urban expansion. The northeast, as the old industrial base of China, has long relied on mineral resources for development. In recent years, with the exhaustion of resources and the emergence of excess capacity, cities have been forced to adjust their industrial structures. However, such adjustment has been sluggish, and new economic growth points have not been fostered. As a result, many people lost their jobs and left the region, resulting in the emergence of shrinking cities. The central and western regions are relatively closed geographically and backward in their economic development. A large number of laborers thus migrated out to seek employment opportunities, and urban populations gradually shrunk.

Growing and shrinking cities have different carbon-emission characteristics. The emissions of RGCs show a trend of fluctuating growth, while RSCs present an inverted U-shaped trend. SGCs and SSCs show a trend of first rising and then developing steadily. This differs from Xiao et al. [30] but supports Qiang et al. [42], namely, that air pollution is reduced in shrinking cities. RGCs have developed economies and high urbanization levels, which all contribute to increases in carbon emissions. At the same time, the rapid development of transportation and tertiary industry also increase emissions. As for RSCs, most depend on heavy industry. With the exhaustion of resources and the emergence of overcapacity, cities have been forced to reduce the scale of secondary industry, causing urban economies to decline rapidly, which correspondingly reduces emissions. The economic growth of SGCs and SSCs is relatively flat, and technology is improving, causing emissions to tend to be stable.

The results indicate that economic development and population growth positively affect emissions in the four city groups, while technological progress has a negative effect. The proportion of tertiary industry positively affects emissions in growing cities and negatively affects them in shrinking cities. Supporting Zheng et al. [43], economic development and population growth both increase energy consumption and therefore play obvious roles in increasing emissions. Technological advancement can produce more output using less energy, which reduces carbon emissions. The reason the industrial structure of different groups has different effects on emissions might be related to tertiary industry development. If tertiary industry is fully developed, services such as urban transport will require more energy, contributing to higher emissions. However, if urban tertiary industry development is immature, tertiary industry will consume less energy than secondary industry, which will reduce emissions.

## 5. Conclusions and Policy Implications

Aiming to support emission-reduction policy making, this study investigates the characteristics of and factors affecting carbon emissions in growing and shrinking Chinese cities. Taking 280 cities as samples, a UDD index developed by the authors was used to divide the cities into four groups: RGCs, SGCs, RSCs, and SSCs. Emission characteristics are discussed in terms of economics, population, energy intensity, and industry structure. The main findings are summarized below.

For RGCs, their economies and populations grow rapidly, their industrial transformation takes place early, and their tertiary industry development is sufficient. Correspondingly, carbon emissions show a fluctuating growth trend. The regression results show that the proportion of tertiary industry has a significant positive effect on carbon emissions, while other factors have no significant effects. For RSCs, economic growth is declining, population loss occurs with the decline of secondary industry, and industrial structure adjustment is belated. Their carbon emissions show an inverted U shape. The regression results show that economics and population have significant positive effects on emissions, while technology and the proportion of tertiary industry have significant negative effects. For SGCs, their economies and populations have both grown steadily, and tertiary industry has developed continuously with the optimization of industrial structure. Their carbon emissions first rise and then develop steadily. The regression results show that economics and population have significant positive effects on emissions, while technology has a significant negative effect. For SSCs, the level of economic development is low but continues to grow, and the population tends to decrease. Their carbon emissions are similar to those of SGCs, first rising and then developing steadily. The regression results show that economics and population have a significant positive effect on emissions, while technology and the proportion of tertiary industry have a significant negative effect.

The policy implications are as follows. For growing cities with sufficient human capital, they can vigorously develop industries with high-added value and low-carbon emissions. Meanwhile, the government should devote more financial funds to improving the level of low-carbon technology. For shrinking cities, future policies should continue to optimize the industrial structure and encourage the development of the tertiary industry. In addition, the government should increase subsidies for high-quality talents to provide sufficient human capital for technological upgrading.

This study has some limitations. First, in the accounting of urban carbon emissions, only energy-related carbon emissions are accounted, and the carbon emissions generated in cement production processes are not accounted. The accounting scopes will be further expanded to improve data quality in future work. Second, in terms of the influencing factors of carbon emissions, this study focuses on social factors, such as population, economy, and technology. In fact, urban growth and shrinkage, as a comprehensive reflection of population, economic, and social changes, have also been proven to have an impact on carbon emissions and environmental conditions [31,42]. In the future, we will further explore the impact of urban growth and shrinkage on carbon emissions.

## Figures and Tables

**Table 1:** Urban development degree indicators selection.

Dimensions	Indicators	Unit
Population	Natural population growth rate	‰
	Total population	10^4^ Person
	Population density	Person/km^2^
Economic	Per capita GDP	Yuan
	Per capita fiscal revenue	Person/yuan
	GDP growth rate	%
Social and Land use	Total retail sales of consumer goods	10^4^ yuan
	Per capita fiscal expenditure	Person/yuan
	Built-up area	Km^2^

**Table 2:** F-test and Hausman test for four city groups.

City Groups	F-Test		Hausman Test	
	F-Value	Prob	Shi-Sq. Statistic	Prob
RGCs	F = 1.07	0.3960	5.29	0.2587
SGCs	F = 54.78	0.0000	193.76	0.0000
RSCs	F = 14.74	0.0000	109.76	0.0000
SSCs	F = 13.77	0.0000	300.80	0.0000

**Table 3:** Regression results.

Explanatory Variables	RGCs	SGCs	RSCs	SSCs
lnA	0.351	0.743 ***	0.68 ***	0.74 ***
lnP	0.449	0.943 ***	0.722 ***	0.605 ***
lnT	−0.424	−0.709 ***	−0.518 ***	−0.547 ***
lnQ	1.337 *	0.042	−0.111 ***	−0.097 ***
C	−6.7864	−12.129	−7.824	−12.55
R-squared	0.2025	0.8912	0.9286	0.9486
F	1.91	972.17	146.13	655.53
Number of obs	90	1260	330	1120

Note: *** *p* < 0.01, * *p* < 0.1.

**Table 4:** Error statistics of actual CO_2_ emissions and estimated values.

Year	RGCs			SGCs			RSCs			SSCs		
	lnCO_2′_	lnCO_2_	Error (%)	lnCO_2′_	lnCO_2_	Error (%)	lnCO_2′_	lnCO_2_	Error (%)	lnCO_2′_	lnCO_2_	Error (%)
2009	8.51	8.86	4.01	7.46	7.43	0.41	8.07	7.78	3.72	6.99	7.00	0.03
2010	8.56	8.89	3.64	7.54	7.52	0.29	8.15	7.86	3.67	7.10	7.09	0.02
2011	8.60	8.96	4.00	7.62	7.62	0.01	8.25	7.99	3.21	7.19	7.19	0.06
2012	8.62	8.96	3.88	7.64	7.65	0.11	8.26	8.00	3.27	7.23	7.22	0.05
2013	8.68	8.94	2.90	7.65	7.66	0.04	8.24	7.96	3.59	7.19	7.21	0.33
2014	8.60	9.26	7.11	7.66	7.66	0.07	8.24	7.93	3.83	7.23	7.21	0.22
2015	8.61	9.00	4.26	7.67	7.68	0.13	8.20	7.89	3.97	7.21	7.20	0.21
2016	8.61	8.95	3.88	7.67	7.67	0.03	8.19	7.84	4.45	7.21	7.18	0.41
2017	8.68	9.06	4.20	7.67	7.67	0.01	8.21	7.88	4.14	7.24	7.22	0.20
2018	8.66	9.10	4.77	7.69	7.71	0.31	8.14	7.90	3.06	7.16	7.22	0.82

## Data Availability

The data presented in this study are available on request from the corresponding author.
